# Phosphodiesterase 4 inhibition but not beta-adrenergic stimulation suppresses tumor necrosis factor-alpha release in peripheral blood mononuclear cells in septic shock

**DOI:** 10.1186/cc7158

**Published:** 2008-12-17

**Authors:** Andreas Link, Simina Selejan, Christoph Maack, Monika Lenz, Michael Böhm

**Affiliations:** 1 Klinik für Innere Medizin III, Universität des Saarlandes, 66421 Homburg/Saar, Germany

## Abstract

**Introduction:**

Stimulation of beta_2_-adrenergic receptors (β_2_-ARs) inhibits tumor necrosis factor-alpha (TNF-α) release in monocytes. In septic shock, endogenous catecholamines induce β_2_-AR downregulation, leading to an increased TNF-α release. The aims of this study were to analyze the molecular mechanisms of β-adrenergic downregulation and to explore therapeutic interventions with maintained anti-inflammatory efficacy in septic shock using the inhibition of phosphodiesterase 4 (PDE4).

**Methods:**

We conducted *in vitro *stimulation of peripheral blood mononuclear cells of healthy volunteers (n = 20) and patients with septic shock (n = 20) with lipopolysaccharide (LPS) or *Staphylococcus aureus *enterotoxin B (SEB) without or with isoprenaline, forskolin (an activator of adenylate cyclase), or ropipram (an inhibitor of PDE4). We also conducted flow cytometric analysis of Toll-like receptor (TLR) 4 and TLR2 surface expression and intracellular TNF-α production of untreated and stimulated CD14^+ ^monocytes. Protein expression of β-ARs, of G proteins, of adenylate cyclase, and of TLRs was measured by Western blotting.

**Results:**

Investigations were done by LPS (100 ng/mL) or SEB (10 ng/mL) when TLR4 and TLR2 were maximally expressed. LPS- or SEB-treated CD14^+ ^monocytes of healthy volunteers were able to produce TNF-α. This effect was attenuated by isoprenaline, forskolin, or rolipram in a concentration-dependent manner. In CD14^+ ^monocytes of patients with septic shock, the anti-inflammatory effect of isoprenaline was completely blunted whereas efficacy of forskolin and rolipram was maintained. CD14^+ ^monocytes of healthy volunteers were compared with patients with septic shock: protein expression of β_2_-ARs was reduced and inhibitory G protein was increased, whereas no changes in adenylate cyclase and stimulatory G protein were found.

**Conclusions:**

In septic shock, the anti-inflammatory effects of catecholamines are blunted by downregulation of β_2_-ARs and upregulation of the inhibitory G protein in CD14^+ ^monocytes. Beta-adrenergic downregulation is overcome by inhibitors of PDE4. These results provide a mechanistic rationale for the therapeutic use of selective PDE4 inhibitors in the treatment of septic shock.

## Introduction

Severe sepsis and septic shock are systemic responses to infection and represent predominant causes of death in intensive care units [1,2], with the incidence of sepsis steadily increasing over the past decades [3]. Monocytes are activated by surface receptors such as Toll-like receptors (TLRs) through recognition of pathogen-derived exogenous ligands: lipopolysaccharides (LPSs), *Staphylococcus aureus *enterotoxin B (SEB), other peptidoglycans, and oligonucleotides. TLR-mediated signaling pathways induce proinflammatory cytokine expression like tumor necrosis factor-alpha (TNF-α) and interleukin-6, triggering the systemic inflammatory response syndrome and the development of septic shock [4,5].

It has long been recognized that β-adrenergic stimulation suppresses LPS-induced TNF-α release from blood cells *in vitro *and *in vivo *[6] and thus could ameliorate the inflammatory response. In severe sepsis and septic shock, neuroendocrine systems are activated. Sympathetic activation is associated with increasing plasma concentrations of epinephrine and norepinephrine [7] and desensitization of the β-adrenergic system (that is, downregulation of β-adrenergic receptor [β-AR] density as shown for the β_1_-ARs in chronic heart failure [8-10]). Also, in patients with septic shock, the hemodynamic response to β_1_-AR agonists was impaired, and in peripheral blood mononuclear cells (PBMCs), a decreased β_2_-AR-mediated cAMP production was found [11-13].

In septic shock, patients require intravenous catecholamines, which may aggravate β-adrenergic desensitization, potentially leading to further deterioration of the hemodynamic situation and overwhelming PBMC TNF-α release. Thus, an attractive therapeutic approach to exert cAMP-mediated anti-inflammatory effects despite β-AR desensitization would be to inhibit phosphodiesterase 4 (PDE4) by rolipram [14,15]. The present study was designed (a) to analyze whether β-AR-mediated inhibition of LPS-induced TNF-α expression is blunted in PBMCs of human patients with septic shock, (b) to clarify which mechanisms of desensitization are involved, and (c) to determine whether the PDE4 inhibitor rolipram is able to suppress LPS-induced TNF-α expression.

## Materials and methods

After approval of the institutional review board and informed consent of all of the study participants were received, blood samples were obtained from 20 healthy volunteers without any signs of infection and from 20 patients with septic shock. Because of age-related differences in leukocyte function, the healthy control group was age-matched (median of 49 years and range of 21 to 75 years). All patients fulfilled criteria of septic shock defined as sepsis with at least one organ dysfunction and the requirement of catecholamine therapy [16]. Demographic and clinical characteristics of patients are presented in Table 1.

**Table 1:** Patients' demographic data, clinical characteristics, and survival

Demographic data	
Age in years, median (range)	52 (25 to 73)
Female/male, number	10/10
Septic shock: reasons	
Pneumonia	8
Urosepsis	4
Cholangitis	4
Endocarditis	3
Catheter-associated	3
Pathogen	
*Staphylococcus epidermidis*, number	6
*Staphylococcus aureus*, number	2
*Haemophilus influenca*, number	4
*Enterococcus faecalis*, number	3
*Escherichia coli*, number	3
*Pseudomonas aeruginosa*, number	2
Severity-of-illness scores	
APACHE II score, median (range)	30 (18 to 52)
SAPS II, median (range)	42 (32 to 66)
Catecholamine therapy	
Dobutamine, number Dose in μg/kg per minute (mean ± SD)	20 (6.4 ± 2.9)
Norepinephrine, number Dose in μg/kg per minute (mean ± SD)	16 (0.2 ± 0.2)
Outcome	
ICU mortality rate, number (percentage)	4 (20)
Hospital mortality rate, number (percentage)	5 (25)

APACHE, Acute Physiology and Chronic Health Evaluation; ICU, intensive care unit; SAPS, Simplified Acute Physiology Score; SD, standard deviation.

### Cell preparation, stimulation, and culture conditions

PBMCs were isolated with the use of a Ficoll density gradient (Biochrom, Berlin, Germany) within 30 minutes after collecting peripheral venous blood from volunteers or septic patients. Cells were cultured at 3 × 10^6 ^cells per milliliter in RPMI 1640 (PAA Laboratories, Cölbe, Germany) supplemented with 5% fetal calf serum (Biochrom), 2 mmol/L glutamine, and antibiotics. Culture media and all reagents were tested for the absence of endotoxins. All incubations were carried out in a humidified incubator at 37°C, 21% O_2 _and 5% CO_2_.

PBMCs were analyzed for (a) changes of TLR4 expression in response to increasing LPS (0.1 to 10,000 ng/mL) and isoprenaline (0.0001 to 10 μmol/L) concentrations and time dependency (6, 12, and 24 hours), (b) changes of TLR2 expression in response to increasing SEB (0.1 to 10,000 ng/mL) and isoprenaline (0.0001 to 10 μmol/L) concentrations and time dependency (6, 12, and 24 hours), (c) changes of LPS-mediated TNF-α expression in the absence or presence of isoprenaline, rolipram, and forskolin (all 0.001 to 10 μmol/L), and (d) changes of SEB-mediated TNF-α expression in the absence or presence of isoprenaline, rolipram, and forskolin (all 0.001 to 10 μmol/L).

### Intracellular cytokine measurement by flow cytometry

For cytokine expression, PBMCs were stimulated with LPS (100 ng/mL) or SEB (10 ng/mL) and reagents simultaneously and incubated for a total of 24 hours. Initially, Brefeldin A (10 μg/mL) (Sigma-Aldrich, Munich, Germany) was added to block extracellular secretion of cytokines. Cell fixation was done with 2 mM EDTA (ethylenediaminetetraacetic acid) for 15 minutes for planned measurement of cytokine expression after 6 hours. Cell membranes were reversibly permeabilized with Saponine (0.1%) (Sigma-Aldrich) in phosphate-buffered saline containing 5% milk powder and 0.1% bovine serum albumin (Sigma-Aldrich). Cell surface markers and intracellular cytokines were labelled with mouse anti-human antibodies conjugated to fluorescent dyes at saturating concentrations in permeabilization buffer. For staining of the cell surface markers of monocytes, we used anti-CD14-PE-Cy5 (anti-CD14-phycoerythrin-cyanine 5), anti-TLR4-FITC (anti-TLR4-fluorescein isothiocyanate), and anti-TLR2-FITC. For staining of intracellular monocyte cytokines, we used anti-TNF-α-FITC. After 45 minutes of incubation at room temperature, cells were washed three times in permeabilization buffer and twice in fluorescence-activated cell sorting (FACS) buffer. Subsequently, stained antigens were fixed with 1% paraformaldehyde.

Measurements were performed on a Becton Dickinson FACScan flow cytometer (Becton Dickinson, Heidelberg, Germany) and the Cellquest software system (Becton Dickinson, Heidelberg, Germany). Monocytes were identified by forward and sideward scatter and CD14 positivity, and 10,000 events of CD14^+ ^monocytes were gated. The data are shown as median fluorescence intensities of TNF-α-producing CD14^+ ^monocytes. This method allows a more complete analysis of the functional activity on a single-cell level compared with conventional methods, as described previously [17].

### Western blotting

Monocytes were isolated from PBMCs by magnetic cell sorting using microbeads in accordance with the instructions of the manufacturer (Dynabeads Monocyte; Invitrogen Corporation, Carlsbad, CA, USA). Isolated monocytes are bead- and antibody-free with a purity of CD14^+ ^monocytes of 95%. These cells were separated in cytosol and membrane proteins, containing equal aliquots of denatured protein (50 μg). Nonspecific binding was blocked by incubation with 5% nonfat dry milk for 1 hour at room temperature. Immunoblotting was performed using the following monoclonal antibodies: β_2_-AR, adenylate cyclase (AC), and α-subunits of stimulatory (G_sα_) and inhibitory (G_iα_) G proteins. Equal loading of total protein was controlled by Poinceau red staining (Serva Electrophoresis, Heidelberg, Germany). The immunoreactive bands were quantified by two-dimensional densitometry using Multianalyst software (Bio-Rad Laboratories, München, Germany).

### Reagents and materials

Standard reagents of the highest available quality were used. LPS (serotype *Escherichia coli *055:B5), SEB (fragment 150–161), isoprenaline, and forskolin were purchased from Sigma-Aldrich. Rolipram from A.G. Scientific (San Diego, CA, USA) was used. The drug concentrations that we used had no cytotoxic influence on the measured PBMCs. The following monoclonal antibodies were used: anti-CD14 PE-Cy5-labelled (clone RMO52, lot 06; Beckman Coulter, Krefeld, Germany), anti-TNF-α FITC-labelled (clone MAb11, lot 28687; BD Pharmingen, San Diego, CA, USA), anti-TLR4 (clone 76B357.1, lot 445414; Abcam, Cambridge, UK), anti-TLR2 (clone T2.5, lot 526240; Abcam), anti-AC (rabbit polyclonal IgG), anti-β_2_-AR (rabbit polyclonal IgG) from Santa Cruz Biotechnology, Inc. (Santa Cruz, CA, USA), anti-G_αs _(rabbit polyclonal IgG) from Upstate Biotechnology (Lake Placid, NY, USA), and anti-G_αi2 _generated as described previously [18].

### Statistics

Data were calculated as mean ± standard error of the mean and were analyzed using one-way analysis of variance. If variances differed significantly, the Mann-Whitney test was used. A *P *value of less than 0.05 was considered significant. Concentrations inducing 50% of the maximal effect (EC_50_) were calculated using GraphPad Prism software (GraphPad Software, Inc., San Diego, CA, USA).

## Results

### Toll-like receptor surface expression on CD14^+ ^monocytes of healthy volunteers

**Figure 1:**
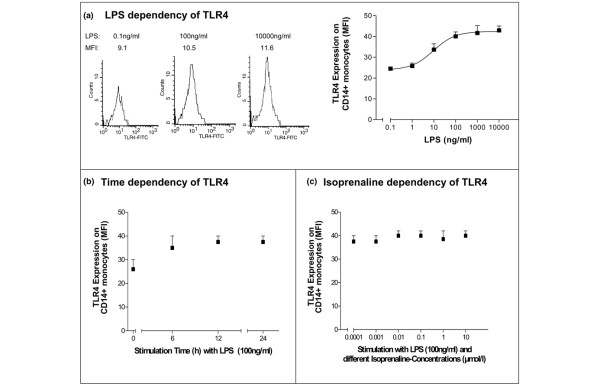
**Modulation of Toll-like-receptor 4 (TLR4) surface expression on CD14^+ ^monocytes of healthy volunteers (n = 20)**. Responses to **(a) **increasing concentrations of lipopolysaccharide (LPS), **(b) **time dependency under continuous LPS stimulation (100 ng/mL), and **(c) **increasing concentrations of isoprenaline are shown. TLR4 expression was measured by flow cytometry, and histograms of median fluorescence intensity (MFI) and averages with standard error of the mean from all probands are shown. FITC, fluorescein isothiocyanate.

**Figure 2:**
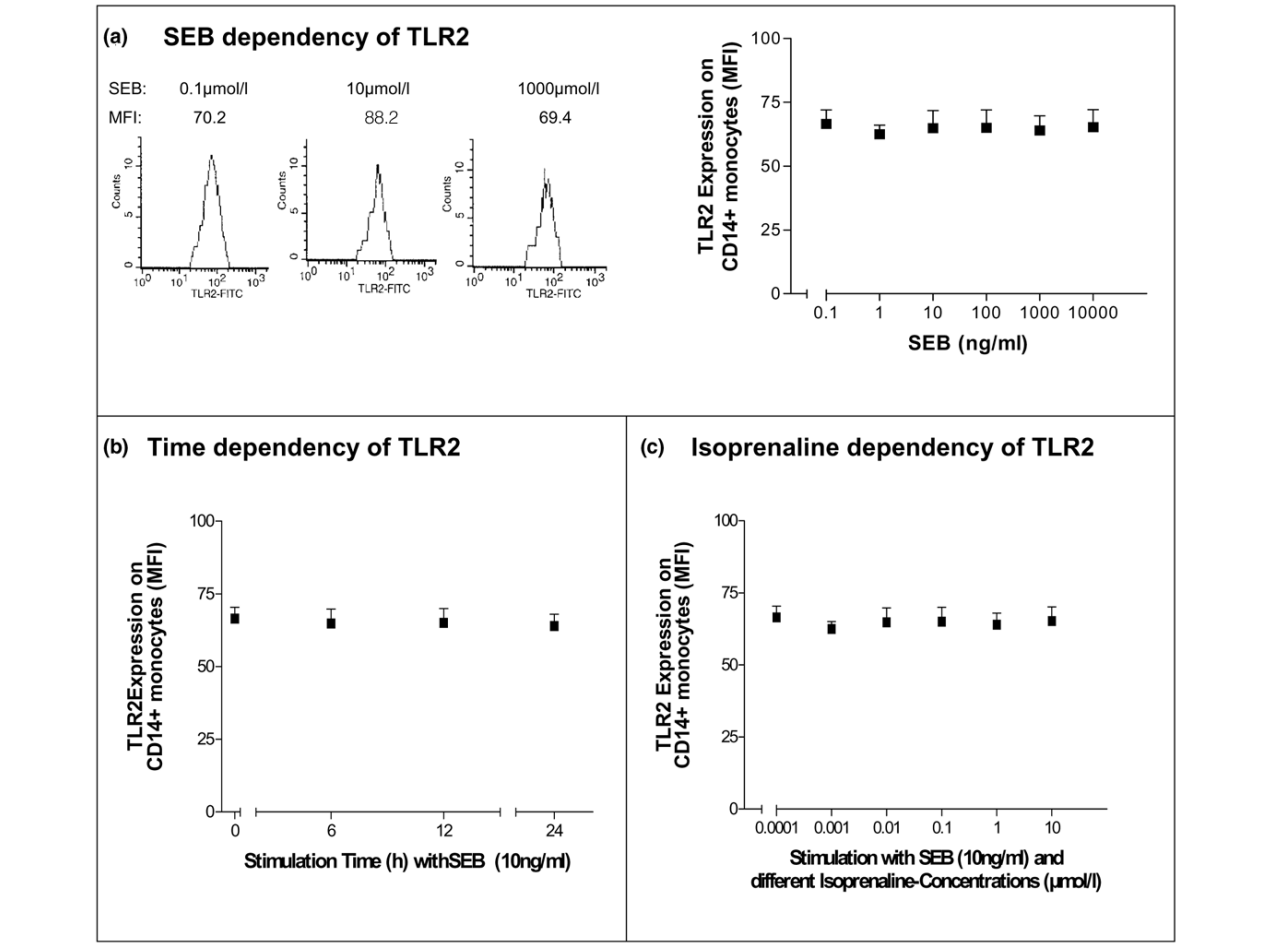
**Modulation of Toll-like-receptor 2 (TLR2) surface expression on CD14^+ ^monocytes of healthy volunteers (n = 10)**. Responses to **(a) **increasing concentrations of *Staphylococcus aureus *enterotoxin B (SEB), **(b) **time dependency under continuous SEB stimulation (10 ng/mL), and **(c) **increasing concentrations of isoprenaline are shown. TLR2 expression was measured by flow cytometry, and histograms of median fluorescence intensity (MFI) and averages with standard error of the mean from all probands are shown. FITC, fluorescein isothiocyanate.

To investigate whether TLRs could participate in the activation of normal monocytes, we analyzed TLR4 and TLR2 expression on CD14^+ ^monocytes after stimulation with increasing doses of LPS or SEB. Cytometric analysis of TLR4 and TLR2 protein expression of untreated monocytes revealed a constitutive expression of both receptors (Figures 1 and 2). Treatment with LPS induced a significant upregulation of TLR4 expression even at low LPS concentrations, achieving a submaximal expression at 100 ng/mL (Figure 1a). CD14^+ ^monocytes treated with increasing LPS concentrations had stimulatory effects of the TLR4 surface expression with an EC_50 _value of 10 ng/mL. Therefore, all further investigations were done by 100 ng/mL LPS when TLR4 was maximally expressed. During a continuous LPS stimulation (100 ng/mL), TLR4 expression was significantly increased within 6 hours and the expression remained stable between 6 and 24 hours (Figure 1b). Treatment with isoprenaline did not alter the expression of TLR4 surface expression (Figure 1c).

Treatment with SEB induced no relevant changes of TLR2 expression even at low and high concentrations (Figure 2a). All further investigations were done by 10 ng/mL SEB when TLR2 was maximally expressed. During a continuous SEB stimulation (10 ng/mL), TLR2 expression remained unchanged between 6 and 24 hours (Figure 2b). Treatment with isoprenaline did not alter the expression of TLR2 surface expression (Figure 2c).

### Inhibition of LPS-/SEB-mediated tumor necrosis factor-alpha expression in CD14^+ ^monocytes of healthy volunteers

**Figure 3:**
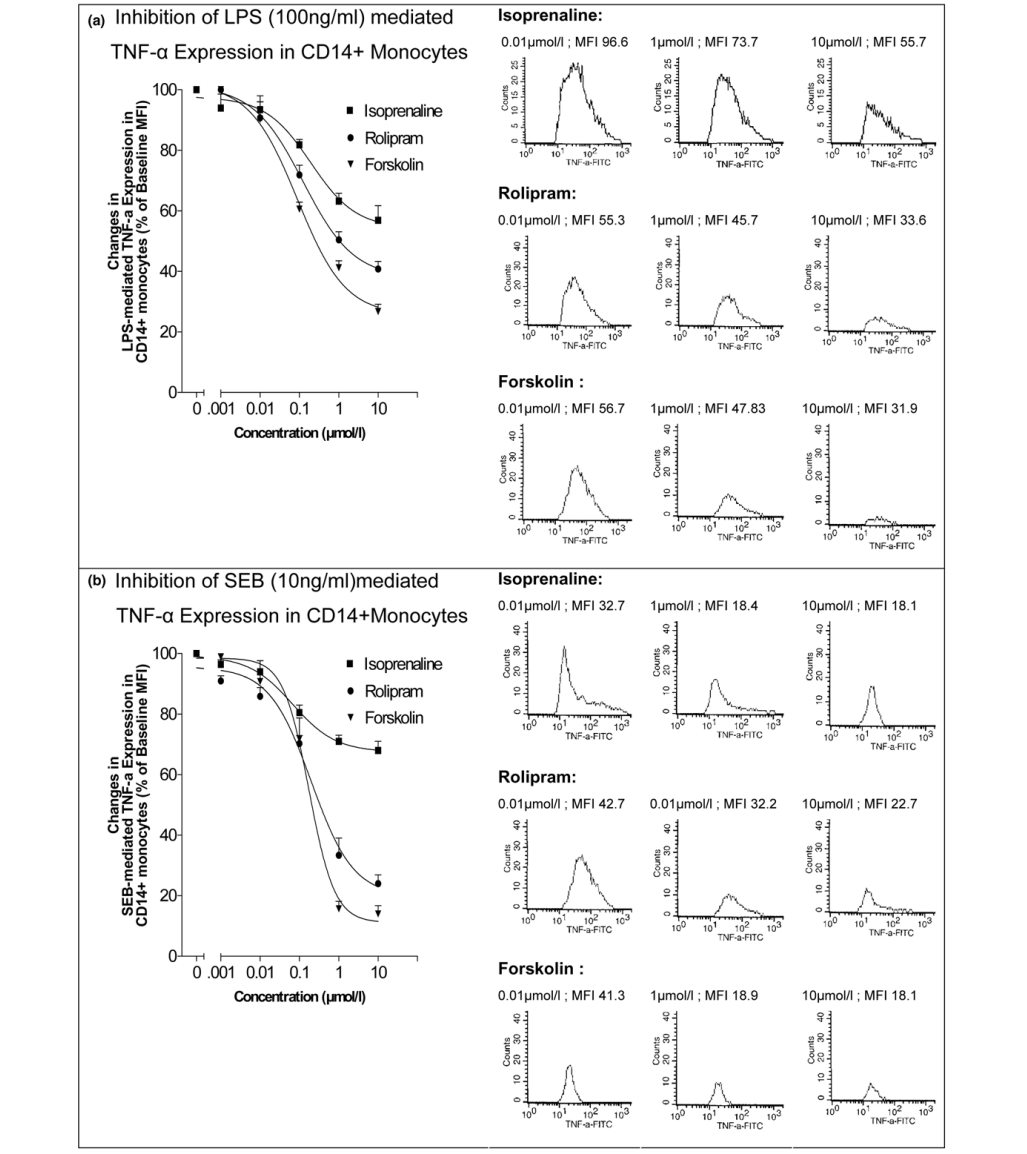
**Suppression of lipopolysaccharide (LPS)-mediated or *Staphylococcus aureus *enterotoxin B (SEB)-mediated tumor necrosis factor-alpha (TNF-α) expression in CD14^+ ^monocytes of healthy volunteers**. Suppression of LPS-mediated (100 ng/mL) **(a) **or SEB-mediated (10 ng/mL) **(b) **TNF-α expression in CD14^+ ^monocytes of healthy volunteers (n = 20) in a concentration-dependent manner by isoprenaline, rolipram, and forskolin (all 0.001 to 10 μmol/L) is shown. TNF-α expression was measured by flow cytometry, and histograms of median fluorescence intensity (MFI) and averages with standard error of the mean from all probands are shown. FITC, fluorescein isothiocyanate.

Incubation of PBMCs from healthy volunteers with LPS for 24 hours increased the expression of TNF-α in a concentration-dependent manner. At 100 ng/mL of LPS, 72.3% ± 3.5% of CD14^+ ^monocytes produced TNF-α. Nonselective β-AR stimulation with isoprenaline (coincubation with 1 μmol/L) suppressed this maximal LPS-induced TNF-α expression up to 40% ± 4.5%. This inhibitory effect of isoprenaline on TNF-α expression was concentration-dependent with an EC_50 _value of 0.1 μmol/L. Also, the selective inhibition of PDE4 with rolipram suppressed TNF-α expression up to 58% ± 4.5% and the direct activation of AC with forskolin suppressed TNF-α expression up to 75% ± 3.8% (Figure 3a).

Incubation of PBMCs from healthy volunteers with SEB for 24 hours increased the expression of TNF-α in a concentration-dependent manner. At 10 ng/mL of SEB, 75.1% ± 3.5% of CD14^+ ^monocytes produced TNF-α. Nonselective β-AR stimulation with isoprenaline (1 μmol/L) suppressed this maximal SEB-induced TNF-α expression up to 25% ± 4.5%. Also, the selective inhibition of PDE4 with rolipram suppressed TNF-α expression up to 75% ± 5.5% and the direct activation of AC with forskolin suppressed TNF-α expression up to 85% ± 4.5% (Figure 3b).

### Beta-adrenergic downregulation on CD14^+ ^monocytes in patients with septic shock

**Figure 4:**
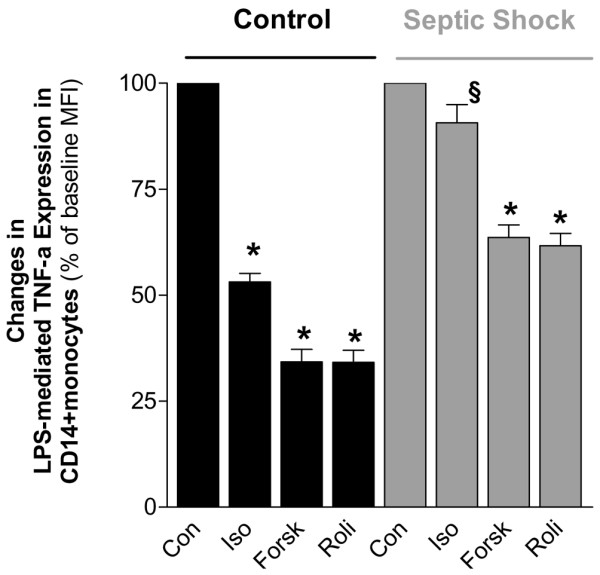
**Lipopolysaccharide (LPS)-induced tumor necrosis factor-alpha (TNF-α) expression in CD14^+ ^monocytes of patients with septic shock and healthy controls**. LPS-induced (100 ng/mL) TNF-α expression in CD14^+ ^monocytes of patients with septic shock (n = 20) and healthy controls (n = 20) in the absence (Con) and presence of isoprenaline (Iso), forskolin (Forsk), or rolipram (Roli) (all 1 μmol/L). *P < 0.05 versus respective control. ^§^*P *< 0.05 versus Iso in healthy volunteers group.

**Figure 5:**
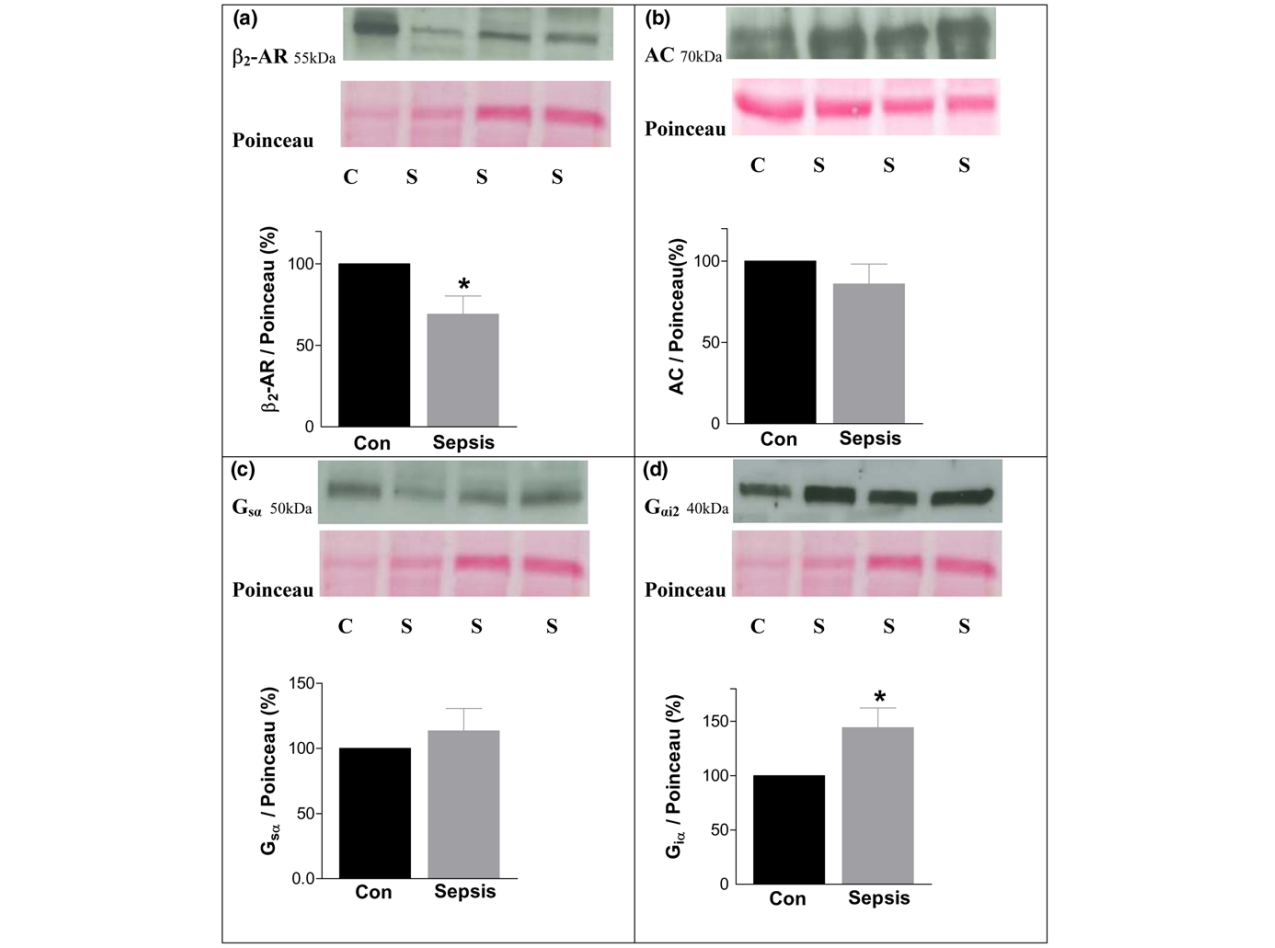
**CD14^+ ^monocytes of patients with septic shock (S) (n = 20) compared with healthy controls (C) (n = 20)**. Immunoblot analysis of protein expression and equal loading of total protein controlled by Poinceau red staining and densitometric quantification are shown. **(a) **Beta_2_-adrenergic receptors (β_2_-ARs) of membranes, **(b) **adenylate cyclase (AC) of cytosol, **(c) **α-subunits of stimulatory G protein (G_sα_) of membranes, and **(d) **α-subunits of the inhibitory G protein (G_iα_) of membranes are shown. * *P *< 0.001 versus control. Con, control.

Detailed patient characteristics and critical illness scores are presented in Table 1. All patients received intravenous catecholamines for hemodynamic stabilization. The LPS-mediated TNF-α expression in CD14^+ ^monocytes of these patients could not be suppressed by isoprenaline, while forskolin and rolipram inhibited the TNF-α expression in these CD14^+ ^monocytes (n = 20, *P *< 0.001, Figure 4). These data are an indirect piece of evidence that β-ARs could be downregulated in CD14^+ ^monocytes of patients with septic shock compared with healthy controls. To elucidate the detailed mechanism of β-adrenergic downregulation, Western blot analysis of β_2_-ARs, AC, and G_sα _and G_iα _were performed. In CD14^+ ^monocytes of patients with septic shock, protein expression of β_2_-ARs was downregulated by 31% ± 11% (*P *< 0.001) whereas expression of G_iα _was upregulated by 44% ± 17% (*P *< 0.001) compared with healthy controls (Figure 5). In contrast, expressions of G_sα _and AC were unchanged.

## Discussion

The main findings of the study are that, in CD14^+ ^monocytes of patients with septic shock, downregulation of β_2_-ARs and upregulation of G_iα _induce β-adrenergic desensitization. This blunts the catecholamine-induced suppression of LPS-induced or SEB-induced TNF-α release and thus may aggravate the proinflammatory response in septic shock. Since *ex vivo *incubation with LPS and isoprenaline reproduced β-adrenergic desensitization in monocytes of healthy volunteers, elevated plasma concentrations of catecholamines and LPS may contribute to β-adrenergic desensitization in these patients *in vivo*. Furthermore, the anti-inflammatory response of the PDE4 inhibitor rolipram was maintained, providing a mechanistic rationale for the use of PDE4 inhibitors in PBMCs of patients with septic shock.

In agreement with previous studies [6,12], β-adrenergic stimulation suppressed the LPS-induced TNF-α expression in a concentration-dependent manner in CD14^+ ^monocytes of healthy volunteers. This effect was mediated by β_2_-ARs, but not β_1_-ARs, confirming previous observations in human macrophages [19]. Similarly, direct activation of AC by forskolin or inhibition of PDE4 with rolipram reduced LPS-induced TNF-α expression.

An important observation of the present study is that, in CD14^+ ^monocytes from patients with septic shock, β-AR-mediated suppression of TNF-α expression was completely abrogated. Previously, Bergmann and colleagues [12] observed that, in the whole blood of patients with septic shock, the epinephrine-induced suppression of LPS-mediated TNF-α release was reduced by only approximately 50% compared with healthy volunteers. The reason for this discrepancy remains unclear. The use of epinephrine or norepinephrine instead of isoprenaline is unlikely to account for this difference since it has been shown previously that epinephrine-induced stimulation of α-ARs does not mediate the suppression of TNF-α release [6]. However, while in our study the use of FACS analysis allowed the estimation of TNF-α expression specifically in CD14^+ ^monocytes, the study of Bergmann and colleagues [12] assessed TNF-α release into the blood from a large variety of potential sources without discrimination of the underlying cell types. Thus, in their study, sources other than monocytes may have contributed to residual suppression of LPS-induced TNF-α release.

Previous studies have not fully resolved the precise mechanisms for β-adrenergic desensitization of the anti-inflammatory effect of catecholamines in septic shock. Bernardin and colleagues [13] observed that, in PBMCs of patients with severe sepsis, production of isoprenaline-, NaF-, and forskolin-induced cAMP was each substantially blunted, suggesting heterologous desensitization of the β-adrenergic pathway with defects at the level of AC. In contrast, we did not observe any changes in the protein expression of AC or G_*s*_, but downregulation of β_2_-AR and upregulation of G_iα_. Upregulation of G_iα _is in agreement with our previous observation of upregulated G_iα _expression in human myocardium from patients with septic shock [10]. Importantly, the changed expression of β-adrenergic signaling components was in concert with the functional effects since both forskolin- and rolipram-induced suppressions of LPS-induced TNF-α release were maintained. These data clearly argue against defects at the level of AC in PBMCs of patients with septic shock, as suggested by Bernardin and colleagues [13].

During septic shock, LPS-mediated or SEB-mediated TNF-α release and elevated catecholamines aggravate β-adrenergic desensitization, and a vicious cycle of unopposed (catecholamine-refractory) inflammation may develop. The mechanisms of β-adrenergic downregulation in septic shock are of particular relevance for the development of therapeutic strategies. In animal models of septic shock, treatment with the PDE4 inhibitor rolipram or transgenic ablation of PDE4B [15] ameliorated the inflammatory response of PBMCs and improved survival. However, PDE4 inhibitors have not been tested in humans with septic shock yet, and results from animal models may not be readily extrapolated to the human situation. According to the data of Bernardin and colleagues [13], who found heterologous desensitization at the level of AC, one could have expected reduced anti-inflammatory efficacy of rolipram in PBMCs of septic patients. Conversely, in our study, unchanged AC expression and maintained anti-inflammatory efficacy of rolipram provide a mechanistic rationale for the use of PDE4 inhibitors in patients with septic shock. Our findings are limited by missing data about levels of PDE4 (or PDE4 activity) in CD14^+ ^monocytes from control subjects and patients with sepsis; these data would strengthen the conclusion that inhibition of PDE4 activity is a potential treatment for sepsis. The clinical use of PDE4 inhibitors is currently limited to patients with depression [20], chronic obstructive pulmonary disease [21,22], and allergic asthma [23].

## Conclusion

This study demonstrates that, in septic shock, the anti-inflammatory effects of catecholamines are blunted by downregulation of β_2_-ARs and upregulation of the inhibitory G proteins in CD14^+ ^monocytes. Beta-adrenergic downregulation is overcome by inhibitors of PDE4. These results provide a mechanistic rationale for the therapeutic use of selective PDE4 inhibitors in the treatment of septic shock. Trials to evaluate the efficacy of PDE4 inhibition in septic shock are warranted.

## Key messages

• Anti-inflammatory effects of catecholamines are blunted by downregulation of beta_2_-adrenergic receptors (β_2_-ARs) and upregulation of the inhibitory G proteins in CD14^+ ^monocytes in septic shock.

• Beta-adrenergic downregulation is overcome by inhibitors of phosphodiesterase 4 (PDE4).

• Anti-inflammatory effects of PDE4 inhibitors are maintained in β-AR downregulated monocytes in septic shock.

• PDE4 inhibition could be a new therapeutic strategy in the treatment of septic shock.

## Abbreviations

β-AR: beta-adrenergic receptor; AC: adenylate cyclase; Cy5: cyanine 5; EC_50_: 50% of the maximal effect; FACS: fluorescence-activated cell sorting; FITC: fluorescein isothiocyanate; G_iα_: alpha-subunit of the inhibitory G protein; G_sα_: alpha-subunit of stimulatory G protein; LPS: lipopolysaccharide; PBMC: peripheral blood mononuclear cell; PDE4: phosphodiesterase 4; PE: phycoerythrin; SEB: *Staphylococcus aureus *enterotoxin B; TLR: Toll-like receptor; TNF-α: tumor necrosis factor-alpha.

## Competing interests

The authors declare that they have no competing interests.

## Authors' contributions

AL coinitiated the study, participated in experimental investigations and in statistical analysis of the data and interpretation of the data, and drafted the manuscript. MB coinitiated the study. SS and ML participated in experimental investigations. CM participated in statistical analysis of the data and interpretation of the data. All authors read and approved the final manuscript.

## References

[B1] Parrillo JE, Parker MM, Natanson C, Suffredini AF, Danner RL, Cunnion RE, Ognibene FP (1990). Septic shock in humans. Advances in the understanding of pathogenesis, cardiovascular dysfunction, and therapy. Ann Intern Med.

[B2] Wheeler AP, Bernard GR (1999). Treating patients with severe sepsis. N Engl J Med.

[B3] Martin GS, Mannino MD, Eaton S, Moss M (2003). The epidemiology of sepsis in the United States from 1979 through 2000. N Engl J Med.

[B4] Muzio M, Polentarutti N, Bosisio D, Prahladan MK, Mantovani A (2000). Toll-like receptors: a growing family of immune receptors that are differentially expressed and regulated by different leukocytes. J Leukoc Biol.

[B5] Aderem A, Ulevitch RJ (2000). Toll-like receptors in the induction of the innate immune response. Nature.

[B6] Lin WJ, Yeh WC (2005). Implication of Toll-like receptor and tumor necrosis factor alpha signaling in septic shock. Shock.

[B7] Annane D, Trabold F, Sharshar T, Jarrin I, Blanc AS, Raphael JC, Gajdos P (1999). Inappropriate sympathetic activation at onset of septic shock: a spectral analysis approach. Am J Respir Crit Care Med.

[B8] Bristow MR, Ginsburg R, Minobe W, Cubicciotti RS, Sageman WS, Lurie K, Billingham KE, Harrison DC, Stinson EB (1982). Decreased catecholamine sensitivity and beta-adrenergic-receptor density in failing human hearts. N Engl J Med.

[B9] Ungerer M, Böhm M, Elce JS, Erdmann E, Lohse MJ (1993). Altered expression of beta-adrenergic receptor kinase and beta 1-adrenergic receptors in the failing human heart. Circulation.

[B10] Böhm M, Kirchmayr R, Gierschik P, Erdmann E (1995). Increase of myocardial inhibitory G-proteins in catecholamine-refractory septic shock or in septic multiorgan failure. Am J Med.

[B11] Silverman HJ, Penaranda R, Orens JB, Lee NH (1993). Impaired beta-adrenergic receptor stimulation of cyclic adenosine monophosphate in human septic shock: association with myocardial hyporesponsiveness to catecholamines. Crit Care Med.

[B12] Bergmann M, Gornikiewicz A, Sautner T, Waldmann E, Weber T, Mittlbock M, Roth E, Fugger R (1999). Attenuation of catecholamine-induced immunosuppression in whole blood from patients with sepsis. Shock.

[B13] Bernardin G, Strosberg AD, Bernard A, Mattei M, Marullo S (1998). Beta-adrenergic receptor-dependent and -independent stimulation of adenylate cyclase is impaired during severe sepsis in humans. Intensive Care Med.

[B14] Prabhakar U, Lipshutz D, Bartus JO, Slivjak MJ, Smith EF, Lee JC, Esser KM (1994). Characterization of cAMP-dependent inhibition of LPS-induced TNF alpha production by rolipram, a specific phosphodiesterase IV (PDE IV) inhibitor. Int J Immunopharmacol.

[B15] Jin SL, Lan L, Zoudilova M, Conti M (2005). Specific role of phosphodiesterase 4B in lipopolysaccharide-induced signaling in mouse macrophages. J Immunol.

[B16] (1992). American College of Chest Physicians/Society of Critical Care Medicine Consensus Conference: definitions for sepsis and organ failure and guidelines for the use of innovative therapies in sepsis. Crit Care Med.

[B17] Link A, Ayadhi T, Böhm M, Nickenig G (2006). Rapid immunomodulation by rosuvastatin in patients with acute coronary syndrome. Eur Heart J.

[B18] Böhm M, Larisch K, Erdmann E, Camps M, Jakobs K, Gierschik P (1991). Failure of [32P]ADP-ribosylation by pertussis toxin to determine Gi alpha content in membranes from various human tissues. Improved radioimmunological quantification using the 125I-labelled C-terminal decapeptide of retinal transducin. Biochem J.

[B19] Izeboud CA, Mocking JA, Monshouwer M, van Miert AS, Witkamp RF (1999). Participation of beta-adrenergic receptors on macrophages in modulation of LPS-induced cytokine release. J Recept Signal Transduct Res.

[B20] Hebenstreit GF, Fellerer K, Fichte K, Fischer G, Geyer N, Meya U, Sastre-y-Hernandez M, Schony W, Schratzer M, Soukop W (1989). Rolipram in major depressive disorder: results of a double-blind comparative study with imipramine. Pharmacopsychiatry.

[B21] Rabe KF, Bateman ED, O'Donnell D, Witte S, Bredenbroker D, Bethke TD (2005). Roflumilast – an oral anti-inflammatory treatment for chronic obstructive pulmonary disease: a randomised controlled trial. Lancet.

[B22] Calverley PM, Sanchez-Toril F, McIvor A, Teichmann P, Bredenbroeker D, Fabbri LM (2007). Effect of one year treatment with roflumilast in severe chronic obstructive pulmonary disease. Am J Respir Crit Care Med.

[B23] Timmer W, Leclerc V, Birraux G, Neuhauser M, Hatzelmann A, Bethke T, Wurst W (2002). The new phosphodiesterase 4 inhibitor roflumilast is efficacious in exercise-induced asthma and leads to suppression of LPS-stimulated TNF-alpha *ex vivo*. J Clin Pharmacol.

